# 

**Published:** 1887-10

**Authors:** 


					﻿OCTOBER, 1887’-
OLD SERIES
VoL XXV
AJaL,s^
& 1855 O
NEW SERIE8
Vol. IV.—No, 8.'
The Atlanta
Medical and Surgical
Journal

Edited by
' W.F.WESTMORELAND, NB
H.V.M.M1LLER. M.D.LL ,D.l
(■JAMES A.GRAY, MJ).
-0UBL1SHED BY JAMES RHARRISQN&Coj
ffi.	SiST LANTA, GA. AH
SUBSCRIPTION: S2.6O A YEAR.
				

